# Some Health Principles

**Published:** 1857-08

**Authors:** 


### SOME HEALTH PRINCIPLES.

No small share of disease and suffering is owing to the error of one man making another's experience a guide for himself, in matters pertaining to bodily health. Nothing can be more true, than that one may do with impunity, what would kill another. We know a lady who will instantly take cold, if she passes across a room or hall which has just been washed. The life of such a person would seem to hang on a very uncertain tenure. But, like a true philosopher, having found that passing through a recently washed room gives her a cold, she simply avoids it, and now, at the full age of three score years and ten, scarcely misses a meal from sickness, in a whole year.

When a man finds out that his constitution is a frail one, his wisest plan is to study its infirmities, to find out its weak points, and like a beleaguered general, the winner of a hundred victories, be always on his guard as to those weak points. An old hat is never made better by being banged about, while by care, it may be made to look *respectable* for years longer. A worn out horse obtains no re-invigoration by hard usage. A man's body, whether frail or strong, is made capable of greater endurance by being well watched over. And take our word for it:

*The best way to harden the constitution is to take care of it.*

That the popular sentiment should prevail that the human constitution is hardened by exposure, when there is nothing like it in the whole range of animated nature, must be classed among the unaccountibilities.

Some men thrive in spite of a given evil habit. This arises from two distinct causes, a vigorous constitution, or from the nature of the human body to adapt itself to the evils of life.

One man may chew tobacco largely, and live to the age of seventy years; another may survive a glass of grog at every meal, or may take a *regular spree* at certain intervals, and yet seem hale and hearty at the end of three score and ten; while a fourth may live equally long, who sits up late and never rises before noon. But it is the utmost of folly to conclude that these are healthy practices, or that they are even innocuous. Multitudes adopt certain habits of life, on the ground that they *are healthy*, because others are healthy who have adopted them, not taking into account the difference of constitution, and least of all, the difference of habits of life. A weakly youth of Fifth Avenue, who *never did a hand's turn in his life*, is placed to feed on pure, rich milk, fresh eggs, and newly-made butter, on the ground that farming people have robust health who eat such things habitually, if indeed, such diet be not a myth in these latitudes. But to have a farmer's health, it is not sufficient that we eat what farmers eat, we must in addition, dress and work as farmers do, otherwise an ordinary farmer's diet will kill off these *half*-imitators in double quick time.

These sentiments will strike the common observer as being justly true, and by taking *the principle of the thing* as our guide, we shall be saved from many dangerous errors in reference to human health. We have of late, seen several pernicious practices inculcated in the newspapers, made pernicious by this glaring want of consideration.

*Cotton stockings* are recommended to be worn in winter, in a recent medical journal, as being more healthful, comfortable, and safe than woolen. The reason the author gives for his advice is, the benefit he derived from the change, corroborated, as he thinks, by the fact that a clergyman who made the change by his counsel, was relieved of a troublesome throat-affection. It would seem incredible that any one of experience should hazard such a rule of health, on such a trifling foundation. The reader has only to try the experiment. Now and then, there may be a person who will be benefitted by the change, but there will be a thousand to be injured by it. We all know that the feet of some are habitually cold, of others dry, of others damp, of others hot, and to recommend cotton stockings in winter, as best for all, or even for a majority, is unpardonable recklessness, or it is the result of inexcusable inexperience. So critical a thing is the change of a covering for the feet, at times, that we never advise any specific change in that respect; we uniformly say, *try both, and adopt that which is most comfortable to yourself.* In our practice as a physician, we strive, at least to give no killing advice, even if we do no good.

To make the illustration more impressive, we give the fact that JOHN FORD, of Auburn, New York, has gone barefooted for two winters. His feet become warmer as colder weather is setting in. He wears shoes in summer, but cannot be induced to do so in winter time. One of the most estimable citizens of our native village, now living, approaching his eightieth year, WILLIAM S. BRYAN, Esq., used to get up on the coldest nights, and walk barefoot over the snow, to cool his feet. The celebrated weatherologist, E. M., writes to the *Journal of Commerce* of the "cold Sunday" night, January 18th, 1857: "Notwithstanding the great refrigerating power of the wind on Brooklyn Heights, when the thermometer was at zero, I continued my observations hourly, throughout the night, clad in my thin, cotton night-dress, with bare feet, except loose slippers, and was at times, five minutes in the open air, without feeling the least sensation of cold."

Mr. Merriam is an old man, in apparent good health; but who does not know that such an exposure would kill half the people in the land? To conclude that such a practice was *healthy*, because it was his habit, and he maintains good health, would be just as wise as to commence drinking liquor habitually, or chewing tobacco, or taking snuff, or any other filthy habit, simply because persons who have persisted in these things have grown old.

This same gentleman writes from Albany, N. Y., a few days later, with the thermometer at thirty degrees below zero.

"I walked three miles this morning in the open air, before sunrise, without any overcoat, and with the same dress I wore in July and August, without feeling cold.

"In the early part of next week, if the roads are passable, I proceed to the Adirondack and other mountains, clad in my Summer costume, and anticipate no difficulty as to cold."

The ability to do such things with impunity, is the result of what is termed an "*Idiosyncrasy*," that is, *something peculiar*, or as Dr. Reese expresses it, with greater scientific accuracy, in his *Medical Lexicon*, now among our standard works, a "*morbid singularity of constitution.*"

It is well known that some crazy people can endure a degree of cold which would destroy healthy life in an hour.

Some persons survive a cold shower-bath of a winter's morning: the same would send multitudes to their grave in a week, and make other multitudes invalids for life. In summing up the whole matter, our advice is,

1st. If you are well, let yourself alone.

2d. If you are not well, make no change in any of your habits without consulting an educated physician.

3d. Do not take it for granted that any habit or practice is advisable for *you*, simply because *others* have health in their observance. But if you want to make the experiment, *first place yourself in all their conditions.*

